# Characteristics and Outcomes Among US Patients Hospitalized for Ischemic Stroke Before vs During the COVID-19 Pandemic

**DOI:** 10.1001/jamanetworkopen.2021.10314

**Published:** 2021-05-17

**Authors:** Adam de Havenon, John P. Ney, Brian Callaghan, Samuel Hohmann, Ernie Shippey, Shadi Yaghi, Mohammad Anadani, Jennifer J. Majersik

**Affiliations:** 1 Department of Neurology, University of Utah, Salt Lake City; 2 Department of Neurology, Boston University, Boston, Massachusetts; 3 Department of Neurology, University of Michigan, Ann Arbor; 4 Research Analytics, Vizient, Irving, Texas; 5 Department of Neurology, New York University, New York; 6 Department of Neurology, Washington University in St Louis, St Louis, Missouri

## Abstract

**Importance:**

After the emergence of COVID-19, studies reported a decrease in hospitalizations of patients with ischemic stroke (IS), but there are little to no data regarding hospitalizations for the remainder of 2020, including outcome data from a large cohort of patients with IS and comorbid COVID-19.

**Objective:**

To assess hospital discharge rates, demographic factors, and outcomes of hospitalization associated with the COVID-19 pandemic among US patients with IS before vs during the COVID-19 pandemic.

**Design, Setting, and Participants:**

This retrospective cohort study used data from the Vizient Clinical Data Base on 324 013 patients with IS at 478 nonfederal hospitals in 43 US states between January 1, 2019, and December 31, 2020. Patients were eligible if they were admitted to the hospital on a nonelective basis and were not receiving hospice care at the time of admission. A total of 41 166 discharged between January and March 2020 were excluded from the analysis because they had unreliable data on COVID-19 status, leaving 282 847 patients for the study.

**Exposure:**

Ischemic stroke and laboratory-confirmed COVID-19.

**Main Outcomes and Measures:**

Monthly counts of discharges among patients with IS in 2020. Demographic characteristics and outcomes, including in-hospital death, among patients with IS who were discharged in 2019 (control group) were compared with those of patients with IS with or without comorbid COVID-19 (COVID-19 and non–COVID-19 groups, respectively) who were discharged between April and December 2020.

**Results:**

Of the 282 847 patients included in the study, 165 912 (50.7% male; 63.4% White; 26.3% aged ≥80 years) were allocated to the control group; 111 418 of 116 935 patients (95.3%; 51.9% male; 62.8% White; 24.6% aged ≥80 years) were allocated to the non–COVID-19 group and 5517 of 116 935 patients (4.7%; 58.0% male; 42.5% White; 21.3% aged ≥80 years) to the COVID-19 group. A mean (SD) of 13 846 (553) discharges per month among patients with IS was reported in 2019. Discharges began decreasing in February 2020, reaching a low of 10 846 patients in April 2020 before returning to a prepandemic level of 13 639 patients by July 2020. A mean (SD) of 13 492 (554) discharges per month was recorded for the remainder of 2020. Black and Hispanic patients accounted for 21.4% and 7.0% of IS discharges in 2019, respectively, but accounted for 27.5% and 16.0% of those discharged with IS and comorbid COVID-19 in 2020. Compared with patients in the control and non–COVID-19 groups, those in the COVID-19 group were less likely to smoke (16.0% vs 17.2% vs 6.4%, respectively) and to have hypertension (73.0% vs 73.1% vs 68.2%) or dyslipidemia (61.2% vs 63.2% vs 56.6%) but were more likely to have diabetes (39.8% vs 40.5% vs 53.0%), obesity (16.2% vs 18.4% vs 24.5%), acute coronary syndrome (8.0% vs 9.2% vs 15.8%), or pulmonary embolus (1.9% vs 2.4% vs 6.8%) and to require intubation (11.3% vs 12.3% vs 37.6%). After adjusting for baseline factors, patients with IS and COVID-19 were more likely to die in the hospital than were patients with IS in 2019 (adjusted odds ratio, 5.17; 95% CI, 4.83-5.53; National Institutes of Health Stroke Scale adjusted odds ratio, 3.57; 95% CI, 3.15-4.05).

**Conclusions and Relevance:**

In this cohort study, after the emergence of COVID-19, hospital discharges of patients with IS decreased in the US but returned to prepandemic levels by July 2020. Among patients with IS between April and December 2020, comorbid COVID-19 was relatively common, particularly among Black and Hispanic populations, and morbidity was high.

## Key Points

**Question:**

What were the hospital discharge rates, demographic factors, and outcomes of hospitalization associated with the COVID-19 pandemic among US patients with ischemic stroke (IS) in 2020?

**Findings:**

In this cohort study of 478 US hospitals with 324 013 patients with IS, substantial decreases in the number of patients discharged with IS were observed at the beginning of the pandemic in February 2020, but these rates returned to prepandemic levels by July 2020. Compared with patients with IS in 2019, those with IS and comorbid COVID-19 in 2020 were less likely to have conventional vascular risk factors or stroke at hospital admission and were more likely to be Black or Hispanic and to experience medical complications and in-hospital death.

**Meaning:**

Among patients with IS in 2020, comorbid COVID-19 was common, especially in Black and Hispanic populations, and in-hospital morbidity and mortality rates were high.

## Teaser

This cohort study uses data from the Vizient Clinical Data Base to examine hospital discharge rates and outcomes of hospitalizations as well as demographic factors among US patients with ischemic stroke before and during the COVID-19 pandemic.

## Introduction

After the emergence of COVID-19, studies reported a decrease in hospital encounters among patients with ischemic stroke (IS).^1,2,3,4,5^ This decrease was contrary to the expectation that rates for IS would remain stable or increase because viral infections, including COVID-19, are factors associated with thromboembolic events.^6,7,8^ Although the burden of COVID-19 increased in the US,^9^ additional data on rates of IS through the end of 2020 have not been published, to our knowledge. Previous research has suggested that patients with IS and comorbid COVID-19 have poor outcomes^10,11,12,13,14,15,16^; however, to our knowledge, that research has not explored the impact of IS being identified at hospital admission vs during hospitalization with COVID-19.^2,17^ This study used a data set from 478 hospitals throughout the US to examine the clinical characteristics and outcomes among 5517 patients with IS and COVID-19 who were discharged in 2020 compared with those of patients with IS who were discharged in 2019.

## Methods

### Data Set

This retrospective cohort study included data from the Vizient Clinical Data Base (CDB), a health care analytics platform used by participating US hospitals for measuring clinical performance, costs, and outcomes.^18^ We included patients who received a diagnosis of IS (based on *International Statistical Classification of Diseases, Tenth Revision, Clinical Modification *[*ICD-10-CM*] codes I63.x and H34.1^19^) who were discharged from 478 hospitals that reported complete patient-level data from January 1, 2019, to December 31, 2020. Patients were eligible for inclusion if they were admitted to the hospital on a nonelective basis and were not receiving hospice care at the time of admission. This study was approved by the University of Utah with a waiver of informed consent because of the study’s retrospective design and use of deidentified data. The study followed the Strengthening the Reporting of Observational Studies in Epidemiology (STROBE) reporting guideline for cohort studies.

### Study Cohorts

All patients with IS who were discharged from the hospital were included in monthly counts. For additional analyses, we excluded patients discharged between January 1 and March 31, 2020, because we could not be certain of their COVID-19 status. We created 3 cohorts of patients with IS: (1) patients with IS who were discharged in 2019 (control group), (2) patients with IS without COVID-19 who were discharged between April 1 and December 31, 2020 (non–COVID-19 group), and (3) patients with IS and laboratory-confirmed COVID-19 who were discharged between April and December 2020 (COVID-19 group). We identified patients with comorbid COVID-19 using *ICD-10-CM* code U07.1, which is reserved for cases that have laboratory confirmation of infection with SARS-CoV-2.^20^ The *ICD-10-CM* U07.1 code was released in late March 2020 and was widely used by April. Although there were other approaches that could have been used to identify COVID-19 cases, these approaches risked misclassification bias because of the absence of laboratory confirmation of SARS-CoV-2 infection.

### Outcomes and Variables

The primary outcome was monthly discharges with ischemic stroke. The secondary outcomes included in-hospital death and favorable discharge, defined as discharge to the home or an acute rehabilitation facility. Medical comorbidities were coded as absent or present based on *ICD-10-CM* codes. Because of data restrictions in the Vizient CDB, patient age could not be reported; thus, we used age categories (18-50 years, 51-64 years, 65-74 years, 75-79 years, and ≥80 years). Race categories (White, Black, Asian, and other or unreported) were self-reported to hospital staff and were independent of Hispanic ethnicity, which was handled as a distinct category. We included race/ethnicity as a variable of interest in the study based on a previous study indicating racial/ethnic disparities among patients with COVID-19.^16^

Use of intravenous (IV) alteplase and endovascular thrombectomy (EVT) for the treatment of acute IS was identified using codes from the *International Classification of Diseases, Tenth Revision, Procedure Coding System* (*ICD-10-PCS*) (codes given in eTable 1 in the Supplement). We also abstracted patient scores from the National Institutes of Health Stroke Scale (NIHSS) (score ranges from 0 to 42, with higher scores indicating greater functional impairment), which was coded as R29.7 in the *ICD-10-CM* in the first available NIHSS data set.^21^ Because NIHSS scores were not available for all patients, analyses including NIHSS data had smaller samples.

### Statistical Analysis

We developed graphical representations of the monthly hospital discharges of patients with IS and the number of IV alteplase and EVT interventions received. For the control, COVID-19, and non–COVID-19 cohorts, we reported descriptive statistics and results of tests for differences between the control group vs the non–COVID-19 group and the non–COVID-19 group vs the COVID-19 group, with a 2-sided *t* test used for interval variables and a χ^2^ test used for binary variables. We used mixed-effects logistic regression models to measure outcomes, with hospital as the clustering variable, to account for potential differences in case mix or hospital resources. Baseline factors were adjusted a priori and comprised patient age, sex, and race/ethnicity; presence of comorbidities, including hypertension, diabetes, dyslipidemia, obesity, atrial fibrillation, smoking, and congestive heart failure; and Elixhauser Comorbidity Index score.^22^

In a sensitivity analysis, we adjusted for NIHSS score and receipt of IV alteplase and EVT to account for stroke severity and interventions that may have impacted the outcomes. We also reported the results of our mixed-effects model of the COVID-19 cohort to assess the association between baseline factors and outcomes.

All analyses were stratified by the presence of IS at hospital admission. If an IS case was not coded as present at the time of admission, it could have been discovered later, either during the course of admission or hospitalization.^23^ For each cohort, we reported the proportion of patients who received acute interventions, died while in the hospital, or had favorable discharges, with each category stratified by the presence of IS at admission. We fit a mixed-effects model with the interaction term *present at admission* × *COVID-19 vs control* to determine whether the presence of IS at admission affected the COVID-19 group more than the control group. All analyses were performed using Stata software, version 16.0 (StataCorp LLC), with significance set at 2-tailed *P* ≤ .05.

## Results

The analysis included 324 013 patients with IS who were discharged from nonfederal US hospitals in 43 states from January 1, 2019, to December 31, 2020. Of 478 hospitals included, 279 (58.4%) were teaching hospitals, 227 (47.5%) had more than 150 beds, and 58 (12.1%) were designated as rural based on US census criteria; 110 hospitals were located in the Northeast census region, 144 in the Midwest, 154 in the South, and 70 in the West.

**Figure 1.**
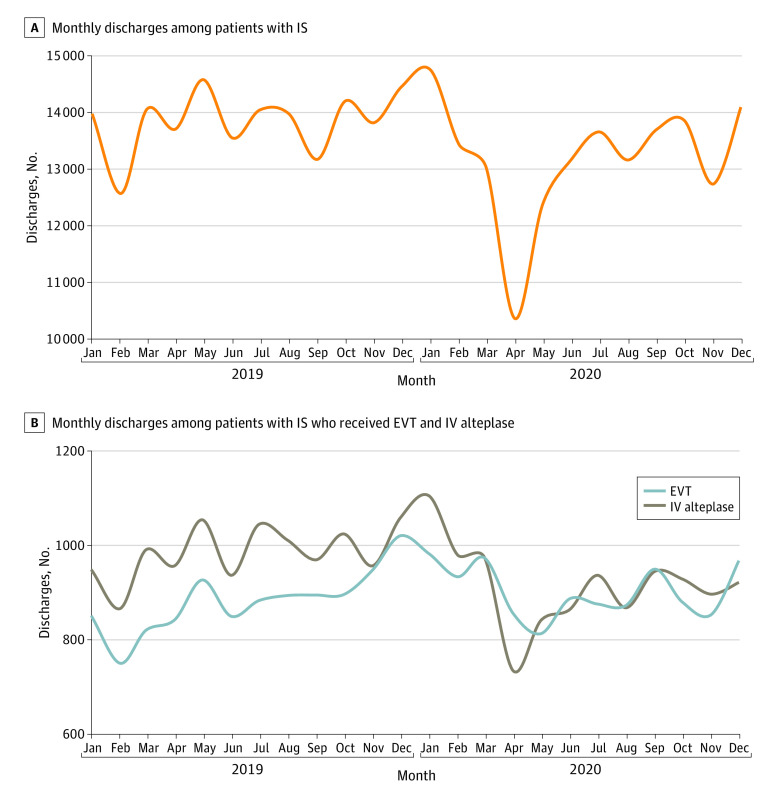
Monthly Hospital Discharges Among Patients With Ischemic Stroke (IS) EVT indicates endovascular thrombectomy; IV, intravenous.

The monthly counts of discharged patients with IS are shown in Figure 1. In 2019, the monthly mean (SD) number of discharges was 13 846 (553); in 2020, the mean (SD) number of monthly discharges was 13 175 (1097). Monthly counts began to decrease in February 2020, reaching a low of 10 846 discharges in April before returning to a prepandemic level of 13 639 discharges by July (Figure 1 and eTable 2 in the Supplement). The mean (SD) number of discharges was 13 492 (554) patients per month for the remainder of 2020. Smaller reductions in the receipt of IV alteplase and EVT also occurred, and these reductions also returned to prepandemic levels by September 2020 (eTable 2 in the Supplement). The monthly count of discharged patients with COVID-19 is shown in the eFigure in the Supplement, with the highest monthly total occurring in December 2020, the last month of the data collection period. The rate of in-hospital death was 6.4% in 2019 and 7.6% in 2020 (*P* < .001).

Among 324 013 total patients, 41 166 discharged between January and March 2020 were excluded from the analysis because they had unreliable data on COVID-19 status. The remaining 282 847 patients were allocated to 3 cohorts: (1) the control group, which comprised 165 912 patients with IS (50.7% male; 63.4% White; 26.3% aged ≥80 years) discharged in 2019; (2) the non–COVID-19 group, which comprised 111 418 of 116 935 patients with IS (95.3%; 51.9% male; 62.8% White; 24.6% aged ≥80 years) discharged between April and December 2020 who did not have COVID-19; and (3) the COVID-19 group, which comprised 5517 of 116 935 patients with IS (4.7%; 58.0% male; 42.5% White; 21.3% aged ≥80 years) discharged between April and December 2020 who had laboratory-confirmed COVID-19.

The demographic characteristics and outcomes in the 3 cohorts are shown in Table 1. Compared with the control group, patients in the non–COVID-19 group were younger (age ≥75 years: 38.2% vs 36.3%); more likely to be male (50.7% vs 51.9%) and smoke (16.0% vs 17.2%); more likely to have obesity (16.2% vs 18.4%), dyslipidemia (61.2% vs 63.2%), intracerebral hemorrhage (6.1% vs 6.9%), and pulmonary embolus (1.9% vs 2.4%); and more likely to be intubated (11.3% vs 12.3%) and experience both favorable discharge (65.9% vs 69.0%) and in-hospital death (6.4% vs 6.8%). However, the magnitude of the differences between these cohorts was relatively small.

**Table 1.** Baseline Demographic Characteristics and Outcomes Among Patients With Ischemic Stroke

Characteristic	Patients^a^				
	Control (n = 165 912)^b^	Non–COVID-19 (n = 111 418)^c^	*P* value^d^	COVID-19 (n = 5517)^e^	*P* value^f^
Age category, y					
<18	752 (0.5)	456 (0.4)	<.001	13 (0.2)	<.001
18-50	18 163 (10.9)	12 692 (11.4)		635 (11.5)	
51-64	42 268 (25.5)	29 373 (26.4)		1531 (27.8)	
65-74	41 344 (24.9)	28 417 (25.5)		1517 (27.5)	
75-79	19 681 (11.9)	13 081 (11.7)		648 (11.7)	
≥80	43 704 (26.3)	27 399 (24.6)		1173 (21.3)	
Age ≥75 y	63 385 (38.2)	40 480 (36.3)	<.001	1821 (33.0)	<.001
Male	84 186 (50.7)	57 849 (51.9)	<.001	3198 (58.0)	<.001
Race/ethnicity^g^					
White	105 168 (63.4)	69 969 (62.8)	.02	2343 (42.5)	<.001
Black	35 477 (21.4)	24 216 (21.7)		1517 (27.5)	
Hispanic	11 674 (7.0)	7850 (7.0)		882 (16.0)	
Asian	4106 (2.5)	2844 (2.6)		195 (3.5)	
Other or unknown	9487 (5.7)	6539 (5.9)		580 (10.5)	
Elixhauser Comorbidity Index score					
Median (IQR)	3 (2-5)	3 (2-5)	<.001	4 (3-6)	<.001
Mean (SD)	3.4 (2.0)	3.6 (2.0)	<.001	4.3 (2.2)	<.001
NIHSS score^h^					
Median (IQR)	5 (2-11)	4 (2-11)	.001	9 (3-16)	<.001
Mean (SD)	7.6 (7.9)	7.5 (7.9)	.04	11.6 (9.6)	<.001
Comorbidities					
Congestive heart failure	38 578 (23.3)	26 243 (23.6)	.07	1460 (26.5)	<.001
Obesity	26 906 (16.2)	20 477 (18.4)	<.001	1349 (24.5)	<.001
Atrial fibrillation	44 865 (27.0)	29 509 (26.5)	.001	1595 (28.9)	.002
Hypertension	121 184 (73.0)	81 466 (73.1)	.66	3765 (68.2)	<.001
Diabetes	66 104 (39.8)	45 110 (40.5)	.001	2925 (53.0)	<.001
Dyslipidemia	101 578 (61.2)	70 368 (63.2)	<.001	3121 (56.6)	<.001
Acute coronary syndrome	13 345 (8.0)	10 230 (9.2)	<.001	874 (15.8)	<.001
Smoking	26 472 (16.0)	19 191 (17.2)	.009	353 (6.4)	<.001
Complications					
Intubation	18 698 (11.3)	13 714 (12.3)	<.001	2077 (37. 6)	<.001
PCI	747 (0.5)	454 (0.4)	.09	21 (0.4)	.45
Intracerebral hemorrhage	10 171 (6.1)	7691 (6.9)	<.001	475 (8.6)	<.001
Subarachnoid hemorrhage	3118 (1.9)	2452 (2.2)	.17	137 (2.5)	.001
Acute kidney failure	32 807 (19.8)	24 555 (22.0)	<.001	2538 (46.0)	<.001
Pulmonary embolus	3132 (1.9)	2636 (2.4)	<.001	376 (6.8)	<.001
Cerebral venous sinus thrombosis	292 (0.2)	254 (0.2)	.002	23 (0.4)	<.001
Deep vein thrombosis	519 (0.3)	380 (0.3)	.20	24 (0.4)	.11
Interfacility transfer	42 161 (25.4)	27 508 (24.7)	<.001	1687 (30.6)	<.001
Presented to emergency department	133 532 (80.5)	90 248 (81.0)	<.001	4160 (75.4)	<.001
Length of hospital stay, mean (SD), d	7.3 (11.9)	7.3 (12.1)	.91	16.2 (20.6)	<.001
Length of ICU stay, mean (SD), d^i^	5.5 (9.5)	5.5 (9.0)	.86	13.4 (15.6)	<.001
Receipt of intervention					
EVT	10 601 (6.4)	7684 (6.9)	<.001	290 (5.3)	.001
IV alteplase	11 847 (7.1)	7675 (6.9)	.049	280 (5.1)	<.001
Outcome					
Favorable discharge	109 258 (65. 9)	76 871 (69.0)	<.001	2115 (38.3)	<.001
In-hospital death	10 636 (6.4)	7544 (6.8)	<.001	1540 (27.9)	<.001

Abbreviations: EVT, endovascular thrombectomy; ICU, intensive care unit; IQR, interquartile range; IS, ischemic stroke; IV, intravenous; NIHSS, National Institutes of Health Stroke Scale; PCI, percutaneous coronary intervention.

^a^ Data are presented as number (percentage) of patients unless otherwise indicated.

^b^ The control group comprised patients with IS who were discharged in 2019.

^c^ The non–COVID-19 group comprised patients with IS who did not have COVID-19 and were discharged between April and December 2020.

^d^ *P* values for non–COVID-19 group vs control group. *P* values were calculated using a χ^2^ test for binary variables, a Wilcoxon rank sum test for ordinal variables, and a *t* test for interval variables.

^e^ The COVID-19 group comprised patients with IS who had COVID-19 and were discharged between April and December 2020.

^f^ *P* values for COVID-19 group vs control group. *P* values were calculated using a χ^2^ test for binary variables, a Wilcoxon rank sum test for ordinal variables, and a *t* test for interval variables.

^g^ All racial categories are non-Hispanic. Races and ethnicities included in the *other* category were not available in the data set.

^h^ A total of 186 243 patients had an NIHSS score available (score ranges from 0 to 42, with higher scores indicating greater functional impairment).

^i^ Length of ICU stay restricted to patients who spent more than 24 hours in the ICU.

The differences in demographic characteristics between the control group and the COVID-19 group were greater. Compared with the control group, patients in the COVID-19 group were younger (age ≥75 years: 38.2% vs 33.0%) and more likely to be male (50.7% vs 58.0%), Black (21.4% vs 27.5%), and Hispanic (7.0% vs 16.0%) (*P* < .001 for all comparisons). With regard to comorbidities, compared with the control group, patients in the COVID-19 group more likely to have obesity (16.2% vs 24.5%) and to have diabetes (39.8% vs 53.0%) but less likely to smoke (16.0% vs 6.4%), have hypertension (73.0% vs 68.2%), and have dyslipidemia (61.2% vs 56.6%) (*P* < .001 for all comparisons). Compared with the control group, medical complications and worse outcomes were also more common in the COVID-19 group, including acute coronary syndrome (8.0% vs 15.8%, respectively), intracerebral hemorrhage (6.1% vs 8.6%), acute kidney failure (19.8% vs 46.0%), pulmonary embolus (1.9% vs 6.8%), intubation (11.3% vs 37.6%), and in-hospital death (6.4% vs 27.9%) (*P* < .001 for all comparisons). Compared with the control group, hospital and intensive care unit stays were more than twice as long in the COVID-19 group (mean [SD] hospital length of stay, 7.3 [11.9] days vs 16.2 [20.6] days; mean [SD] intensive care unit length of stay, 5.5 [9.5] days vs 13.4 [15.6] days), and patients in the COVID-19 group were less likely to receive IV alteplase (7.1% vs 5.1%) and EVT (6.4% vs 5.3%) (Table 1). Among 186 243 discharged patients with an available NIHSS score, those in the COVID-19 group (n = 2314) had significantly higher scores (mean [SD] score, 11.6 [9.6]) compared with those in the control group (n = 92 751; mean [SD] score, 7.6 [7.9]) (*P* < .001).

In the mixed-effects model, the unadjusted odds ratio (OR) of in-hospital death in the COVID-19 group compared with the control group was 5.95 (95% CI, 5.58-6.35). After adjustment for baseline demographic characteristics and comorbidities, the OR was 5.17 (95% CI, 4.83-5.53); after further adjustment for NIHSS score and receipt of IV alteplase and EVT, the OR remained high at 3.57 (95% CI, 3.15-4.05) (Table 2). The unadjusted OR for favorable discharge in the COVID-19 group compared with the control group was 0.31 (95% CI, 0.30-0.33). After adjustment for baseline demographic characteristics and comorbidities, the OR was 0.33 (95% CI, 0.31-0.35); after further adjustment for NIHSS score and receipt of IV alteplase and EVT, the OR was 0.49 (95% CI, 0.44-0.54).

**Table 2.** Mixed-Effects Logistic Regression Analysis of Outcomes^a^

Model	In-hospital death			Favorable discharge		
	OR (95% CI)	SE	*P* value	OR (95% CI)	SE	*P* value
Unadjusted model	5.95 (5.58-6.35)	0.20	<.001	0.31 (0.30–0.33)	0.01	<.001
Adjusted model 1^b^	5.17 (4.83-5.53)	0.18	<.001	0.33 (0.31–0.35)	0.01	<.001
Adjusted model 2^c^	3.57 (3.15-4.05)	0.23	<.001	0.49 (0.44-0.54)	0.03	<.001

Abbreviations: EVT, endovascular thrombectomy; IS, ischemic stroke; IV, intravenous; OR, odds ratio.

^a^ Analysis of 5517 patients with IS and COVID-19 discharged between April and December 2020 compared with control group of 165 912 patients with IS discharged in 2019.

^b^ Adjusted for patient age, sex, and race/ethnicity; presence of diabetes, congestive heart failure, obesity, and smoking; and Elixhauser Comorbidity Index score.

^c^ Adjusted for patient age, sex, and race/ethnicity; presence of diabetes, congestive heart failure, obesity, and smoking; Elixhauser Comorbidity Index score; National Institutes of Health Stroke Scale score; and receipt of IV alteplase and EVT. This model included 2314 patients with IS and COVID-19 discharged between April and December 2020 compared with the control group of 92 751 patients with IS discharged in 2019.

The proportion of patients with IS at admission was slightly higher in the control group (90.5%) than in the non–COVID-19 group (90.1%). However, in the COVID-19 group, IS was present at admission in only 3634 of 5517 patients (65.9%) (Table 3). In the COVID-19 group, among patients without IS at admission (n = 1883) compared with those with IS at admission (n = 3634), the in-hospital death rate was 46.0% vs 18.5% (*P* < .001) and the favorable discharge rate was 20.0% vs 47.8% (*P* < .001).

**Table 3.** Patients With Diagnosis of IS at Hospital Discharge Stratified by Presence of IS Diagnosis at Admission

Variable	Patients, No./total No. (%)				
	Control (n = 165 912)^a^	Non–COVID-19 (n = 111 418)^b^	*P* value^c^	COVID-19 (n = 5517)^d^	*P* value^e^
IS					
Present at admission	150 112/165 912 (90.5)	100 360/111 418 (90.1)	<.001	3634/5517 (65.9)	<.001
Not present at admission	15 800/165 912 (9.5)	11 058/111 418 (9.9)	<.001	1883/5517 (34.1)	<.001
In-hospital death					
IS present at admission	7242/150 112 (4.8)	5018/100 360 (5.0)	.046	673/3634 (18.5)	<.001
IS not present at admission	3394/15 800 (21.5)	2526/11 058 (22.8)	.008	867/1883 (46.0)	<.001
Favorable discharge					
IS present at admission	103 477/150 112 (68.9)	72 487/100 360 (72.2)	<.001	1738/3634 (47.8)	<.001
IS not present at admission	5781/15 800 (36.6)	4384/11 058 (39.6)	<.001	377/1883 (20.0)	<.001
Received IV alteplase					
IS present at admission	11 281/150 112 (7.5)	7304/100 360 (7.3)	.10	200/3634 (5.5)	<.001
IS not present at admission	566/15 800 (3.6)	371/11 058 (3.4)	.37	80/1883 (4.2)	.13
Received EVT					
IS present at admission	9994/150 112 (6.7)	7259/100 360 (7.2)	<.001	254/3634 (7.0)	.43
IS not present at admission	607/15 800 (3.8)	425/11 058 (3.8)	>.99	36/1883 (1.9)	<.001

Abbreviations: EVT, endovascular thrombectomy; IS, ischemic stroke; IV, intravenous.

^a^ The control group comprised patients with IS who were discharged in 2019.

^b^ The non–COVID-19 group comprised patients with IS who did not have COVID-19 and were discharged between April and December 2020.

^c^ *P* values for non–COVID-19 group vs control group.

^d^ The COVID-19 group comprised patients with IS who had COVID-19 and were discharged between April and December 2020.

^e^ *P* values for COVID-19 group vs control group.

In both the control and the non–COVID-19 groups, the rate of in-hospital death was also higher among patients without IS at admission compared with those with IS at admission (control group, 21.5% vs 4.8%; non–COVID-19 group, 22.8% vs 5.0%) (Table 3). Among patients without IS at admission, the rates of favorable discharge were lower than those among patients with IS at admission (control group, 36.6% vs 68.9%; non–COVID-19 group, 39.6% vs 72.2%), as were the receipt of IV alteplase (control group, 3.6% vs 7.5%; non–COVID-19 group, 3.4% vs 7.3%) and EVT (control group, 3.8% vs 6.7%; non–COVID-19 group, 3.8% vs 7.2%). The interaction between the terms *present at admission* × *COVID-19 vs control* was not significant, indicating a higher risk of in-hospital death and unfavorable discharge among patients without IS at admission in both the control and COVID-19 groups. However, the in-hospital mortality rate among patients in the control group without IS at admission was 21.5%; in the COVID-19 group, this rate was more than double at 46.0% (*P* < .001).

**Figure 2.**
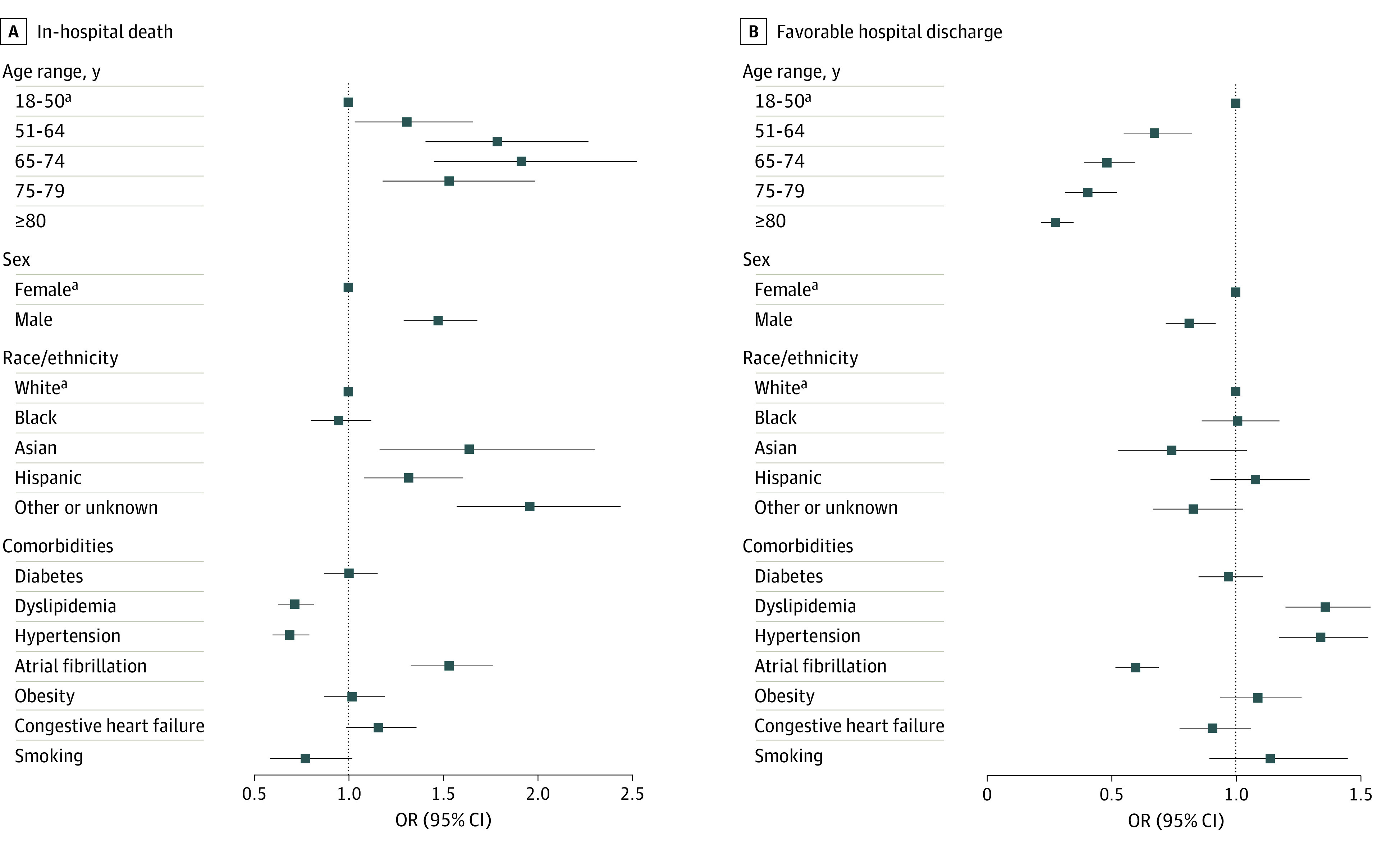
In-Hospital Death and Favorable Hospital Discharge Among Patients With Ischemic Stroke and Comorbid COVID-19 Markers indicate point estimates, with horizontal lines representing 95% CIs. OR indicates odds ratio. ^a^Reference variable.

The ORs in the mixed-effects model for baseline demographic characteristics and comorbidities among the COVID-19 group are shown in Figure 2. Older patients and those who were male, Hispanic, or Asian were more likely to die in the hospital. The presence of atrial fibrillation was also associated with increases in the likelihood of in-hospital death, and smoking and the presence of dyslipidemia and hypertension were associated with a reduced likelihood of in-hospital death. Similar findings in the opposite direction were observed for favorable discharge, with older, male, and Hispanic patients less likely to have favorable discharge; however, the differences were not significant with the exception of older age and male sex.

## Discussion

In this retrospective cohort study, after an initial decrease in IS hospitalizations during the beginning of the COVID-19 pandemic in February 2020, hospitalizations returned to prepandemic levels by July 2020 and stayed at those levels for the remainder of 2020. The reduction in hospitalizations for IS at the beginning of the pandemic may have been associated with patients not seeking medical care owing to fear of acquiring COVID-19. The increase in hospital discharges in the later months of 2020 may have been associated with awareness efforts and/or an easing of public fears regarding hospital care.^4,24^

In adjusted models, patients in the COVID-19 group compared with those in the control group had a 5-fold higher risk of in-hospital death and were 3 times less likely to have a favorable hospital discharge. These associations remained significant in a sensitivity analysis adjusted for stroke severity and interventions. The rate of in-hospital mortality was particularly high (46.0%) among patients in the COVID-19 group who did not have IS at admission. This phenomenon, which could reflect the development of IS during hospitalization or the delayed recognition of simultaneous IS after initial presentation with respiratory symptoms, was more than 3 times as common among patients in the COVID-19 group compared with those in the control group. The higher mortality among those without IS at admission may be associated with the severity of COVID-19 (which may have masked neurologic symptoms and led to deferral of neuroimaging evaluations), a higher prevalence of in-hospital thrombotic complications among those with COVID-19, or ineligibility for acute interventions among inpatients with IS.

Although the incidence of IS and comorbid COVID-19 from April to December 2020 (when it could be accurately measured) was 4.7% among all discharged patients with IS, the association of COVID-19 with in-hospital mortality was observed throughout 2020. In 2019, the in-hospital mortality among discharged patients with IS was 6.4%, and in all of 2020, the in-hospital mortality was 7.6% (*P* < .001). Of note, compared with the control group, patients with IS who did not have laboratory-confirmed COVID-19 and who were discharged between April and December 2020 also had a higher rate of in-hospital death (6.8%) and more complications, such as acute coronary syndrome, pulmonary embolus, and intubation, suggesting that although COVID-19 was not confirmed in these patients, a small percentage of them may have had infection that was not diagnosed or coded as such.

We also found that discharged patients with IS and comorbid COVID-19 were younger and more likely to be male, Black, and Hispanic or have obesity or diabetes but were less likely to smoke or have hypertension or dyslipidemia compared with patients in the control group. These findings are consistent with previous data on COVID-19 demographic factors and disparities.^12,25^ The higher incidence of COVID-19 in Black and Hispanic populations may have been associated with socioeconomic and health disparities that, in turn, may be associated with increases in the risk of developing COVID-19 and IS.^26^ A definitive explanation for the increased risk of COVID-19 among male patients and those with obesity or diabetes has not been found. Whether the higher risk of IS was associated with vascular risk factors that increase susceptibility to more severe COVID-19 or whether the factors were associated with independent or synergistic prothrombotic consequences in patients with COVID-19 is unknown.

Among discharged patients with IS and comorbid COVID-19, we found that the factors associated with in-hospital death were older age; male sex; Asian, Hispanic, and other race/ethnicity; and the presence of atrial fibrillation. Discharged patients with IS and comorbid COVID-19 were also more likely to have medical complications, such as acute coronary syndrome, pulmonary embolus, or intubation, which is consistent with previous findings^12,13^ on the association of COVID-19 with disease severity. In the control group, the mean (SD) NIHSS score was 7.6 (7.9), and in discharged patients with IS and comorbid COVID-19, the mean (SD) score was 11.6 (9.6); this finding suggests that patients with IS and comorbid COVID-19 may experience more severe IS, that COVID-19 may be identified more frequently in patients with severe IS, or that patients with COVID-19 and minor stroke do not seek medical care.

In this cohort study, 65.9% of patients with COVID-19–associated IS presented with IS at admission, meaning that 34.1% of patients did not have IS at admission, which may reflect the inability to recognize IS symptoms in these patients or the occurrence of IS events during hospitalization as a thrombotic complication of COVID-19. Among patients in the control and non–COVID-19 groups, 90.5% and 90.1%, respectively, had IS at admission. Although the rate of in-hospital death was higher among patients in the control group without IS at admission than among those with IS at admission, the comparable rates among patients with COVID-19 were high. For example, the in-hospital death rate among discharged patients without IS at admission in 2019 was 21.5%, whereas it was 46.0% among discharged patients with COVID-19 without IS at admission in 2020. Patients with COVID-19 who did not have IS at admission were also less likely to receive EVT or have favorable discharge, which may represent an opportunity to improve the care of patients with COVID-19 through increased monitoring for IS while in the hospital and/or through strategies to prevent IS.

### Limitations

This study has limitations. The Vizient CDB is not representative of all inpatient discharges in the US, and identification of IS cases through billing codes has the potential for misclassification bias. However, the sample was large, and the data sampling and extraction methods, including those used for hospitals, were consistent across time. We could not capture the severity of COVID-19, which limited our ability to make definitive associations. Apart from the dichotomy of presence or absence of IS at the time of hospital admission, we did not know when the IS event occurred during the course of hospitalization, which prevented us from fully exploring the association between COVID-19 and IS. In addition, the differences between patients with IS in 2019 and patients with IS without COVID-19 in 2020 suggest that some of the patients in the non–COVID-19 group may have had COVID-19 but were not documented as such. Evaluation of longitudinal data with additional variables over subsequent time frames is needed to confirm these findings.

## Conclusions

This cohort study found that after an initial decrease in IS discharges during the beginning of the COVID-19 pandemic in February 2020, discharge counts returned to prepandemic levels by July 2020 and stayed at those levels for the remainder of 2020. In adjusted models, discharged patients with IS and comorbid COVID-19 in 2020 compared with discharged patients with IS in 2019 had a 5-fold higher risk of in-hospital death and were 3 times less likely to have a favorable hospital discharge. We also found that discharged patients with IS and comorbid COVID-19 in 2020 compared with discharged patients with IS in 2019 had more severe stroke and were younger; more likely to be male, Black, and Hispanic; and more likely to have obesity and diabetes but less likely to smoke or have hypertension or dyslipidemia. Among discharged patients with IS and comorbid COVID-19, the factors associated with in-hospital death were older age, male sex, Black or Hispanic race/ethnicity, and atrial fibrillation. The rate of in-hospital death was highest among patients with COVID-19 who did not have IS at admission, which may represent an opportunity to improve the care of patients with COVID-19.

## Notes

Supplement. **eTable 1.** *International Classification of Diseases, Tenth Revision, Procedure Coding System (ICD-10-PCS)* Codes Used for Identification of Selected Study Variables **eTable 2.** Monthly Counts of Patients With Ischemic Stroke and Number Treated With Intravenous Alteplase and Endovascular Thrombectomy **eFigure.** Monthly Rates From April to December 2020 of Patients With Ischemic Stroke and Comorbid COVID-19 and Their Rates of In-Hospital Death

## References

[1] Morelli N, Rota E, Terracciano C, . The baffling case of ischemic stroke disappearance from the casualty department in the COVID-19 era. Eur Neurol. 2020;83(2):213-215. doi:10.1159/000507666 32289789 PMC7179532

[2] Kansagra AP, Goyal MS, Hamilton S, Albers GW. Collateral effect of COVID-19 on stroke evaluation in the United States. N Engl J Med. 2020;383(4):400-401. doi:10.1056/NEJMc2014816 32383831 PMC7233187

[3] Uchino K, Kolikonda MK, Brown D, . Decline in stroke presentations during COVID-19 surge. Stroke. 2020;51(8):2544-2547. doi:10.1161/STROKEAHA.120.030331 32716818 PMC7309646

[4] Lange SJ, Ritchey MD, Goodman AB, . Potential indirect effects of the COVID-19 pandemic on use of emergency departments for acute life-threatening conditions—United States, January-May 2020. MMWR Morb Mortal Wkly Rep. 2020;69(25):795-800. doi:10.15585/mmwr.mm6925e2 32584802 PMC7316316

[5] Nguyen-Huynh MN, Tang XN, Vinson DR, . Acute stroke presentation, care, and outcomes in community hospitals in northern California during the COVID-19 pandemic. Stroke. 2020;51(10):2918-2924. doi:10.1161/STROKEAHA.120.031099 32762619 PMC7434008

[6] Cowan LT, Lutsey PL, Pankow JS, Matsushita K, Ishigami J, Lakshminarayan K. Inpatient and outpatient infection as a trigger of cardiovascular disease: the ARIC study. J Am Heart Assoc. 2018;7(22):e009683. doi:10.1161/JAHA.118.009683 30571501 PMC6404437

[7] Boehme AK, Luna J, Kulick ER, Kamel H, Elkind MSV. Influenza-like illness as a trigger for ischemic stroke. Ann Clin Transl Neurol. 2018;5(4):456-463. doi:10.1002/acn3.545 29687022 PMC5899905

[8] Abou-Ismail MY, Diamond A, Kapoor S, Arafah Y, Nayak L. The hypercoagulable state in COVID-19: incidence, pathophysiology, and management. Thromb Res. 2020;194:101-115. doi:10.1016/j.thromres.2020.06.029 32788101 PMC7305763

[9] Centers for Disease Control and Prevention. COVID data tracker. US Department of Health and Human Services; 2020. Accessed March 8, 2021. https://covid.cdc.gov/covid-data-tracker

[10] Yaghi S, Ishida K, Torres J, . SARS-CoV-2 and stroke in a New York healthcare system. Stroke. 2020;51(7):2002-2011. doi:10.1161/STROKEAHA.120.030335 32432996 PMC7258764

[11] Madjid M, Safavi-Naeini P, Solomon SD, Vardeny O. Potential effects of coronaviruses on the cardiovascular system: a review. JAMA Cardiol. 2020;5(7):831-840. doi:10.1001/jamacardio.2020.1286 32219363

[12] Richardson S, Hirsch JS, Narasimhan M, ; Northwell COVID-19 Research Consortium. Presenting characteristics, comorbidities, and outcomes among 5700 patients hospitalized with COVID-19 in the New York City area. JAMA. 2020;323(20):2052-2059. doi:10.1001/jama.2020.6775 32320003 PMC7177629

[13] Merkler AE, Parikh NS, Mir S, . Risk of ischemic stroke in patients with coronavirus disease 2019 (COVID-19) vs patients with influenza. JAMA Neurol. 2020;77(11):1-7. doi:10.1001/jamaneurol.2020.2730 32614385 PMC7333175

[14] Agarwal S, Scher E, Rossan-Raghunath N, . Acute stroke care in a New York City comprehensive stroke center during the COVID-19 pandemic. J Stroke Cerebrovasc Dis. 2020;29(9):105068. doi:10.1016/j.jstrokecerebrovasdis.2020.105068 32807471 PMC7305900

[15] Nannoni S, de Groot R, Bell S, Markus HS. Stroke in COVID-19: a systematic review and meta-analysis. Int J Stroke. 2021;16(2):137-149. doi:10.1177/1747493020972922 33103610 PMC7859578

[16] de Havenon A, Ney JP, Callaghan B, . Impact of COVID-19 on outcomes in ischemic stroke patients in the United States. J Stroke Cerebrovasc Dis. 2021;30(2):105535. doi:10.1016/j.jstrokecerebrovasdis.2020.105535 33310595 PMC7832426

[17] Markus HS, Brainin M. COVID-19 and stroke—a global World Stroke Organization perspective. Int J Stroke. 2020;15(4):361-364. doi:10.1177/1747493020923472 32310017 PMC11927026

[18] Clinical Data Base. Vizient; 2021. Accessed April 24, 2020. https://www.vizientinc.com/our-solutions/clinical-solutions/clinical-data-base

[19] Chang TE, Tong X, George MG, ; Paul Coverdell National Acute Stroke Program Team. Trends and factors associated with concordance between *International Classification of Diseases, Ninth and Tenth Revision, Clinical Modification* codes and stroke clinical diagnoses. Stroke. 2019;50(8):1959-1967. doi:10.1161/STROKEAHA.118.024092 31208302

[20] World Health Organization. Emergency use *ICD* codes for COVID-19 disease outbreak. World Health Organization; 2020. Accessed June 29, 2020. https://www.who.int/classifications/icd/covid19/en/

[21] Centers for Medicare and Medicaid Services. *ICD-10-CM* Official Guidelines for Coding and Reporting: FY 2019 (October 1, 2018-September 30, 2019). 2019. Accessed January 25, 2021. https://www.cms.gov/Medicare/Coding/ICD10/Downloads/2019-ICD10-Coding-Guidelines-.pdf

[22] Chang HJ, Chen PC, Yang CC, Su YC, Lee CC. Comparison of Elixhauser and Charlson methods for predicting oral cancer survival. Medicine (Baltimore). 2016;95(7):e2861. doi:10.1097/MD.0000000000002861 26886653 PMC4998653

[23] Yu AYX, Holodinsky JK, Zerna C, . Use and utility of administrative health data for stroke research and surveillance. Stroke. 2016;47(7):1946-1952. doi:10.1161/STROKEAHA.116.012390 27174527

[24] Czeisler ME, Marynak K, Clarke KEN, . Delay or avoidance of medical care because of COVID-19–related concerns—United States, June 2020. MMWR Morb Mortal Wkly Rep. 2020;69(36):1250-1257. doi:10.15585/mmwr.mm6936a4 32915166 PMC7499838

[25] Centers for Disease Control and Prevention. COVID-19: people with certain medical conditions. US Department of Health and Human Services. February 11, 2020. Updated March 29, 2021. Accessed June 30, 2020. https://www.cdc.gov/coronavirus/2019-ncov/need-extra-precautions/people-with-medical-conditions.html

[26] McClure ES, Vasudevan P, Bailey Z, Patel S, Robinson WR. Racial capitalism within public health—how occupational settings drive COVID-19 disparities. Am J Epidemiol. 2020;189(11):1244-1253. doi:10.1093/aje/kwaa126 32619007 PMC7337680

