# Supplementary material for: Characteristics and Outcomes Among US Patients Hospitalized for Ischemic Stroke Before vs During the COVID-19 Pandemic
Source: JAMA Netw Open. 2021 May 17;4(5):e2110314. doi: 10.1001/jamanetworkopen.2021.10314 (PMC8129817; doi:10.1001/jamanetworkopen.2021.10314)
Supplement: Supplement. — eTable 1. International Classification of Diseases, Tenth Revision, Procedure Coding System (ICD-10-PCS) Codes Used for Identification of Selected Study Variables eTable 2. Monthly Counts of Patients With Ischemic Stroke and Number Treated With Intravenous Alteplase and Endovascular Thrombectomy eFigure. Monthly Rates From April to December 2020 of Patients With Ischemic Stroke and Comorbid COVID-19 and Their Rates of In-Hospital Death [file jamanetwopen-e2110314-s001.pdf]

Supplementary Online Content

de Havenon A, Ney JP, Callaghan B, et al. Characteristics and outcomes among US patients hospitalized for ischemic stroke before vs during the COVID-19 pandemic. *JAMA Netw Open*. 2021;4(5):e2110314. doi:10.1001/jamanetworkopen.2021.10314

eTable 1. *International Classification of Diseases, Tenth Revision, Procedure Coding System* (*ICD-10-PCS*) Codes Used for Identification of Selected Study Variables

eTable 2. Monthly Counts of Patients With Ischemic Stroke and Number Treated With Intravenous Alteplase and Endovascular Thrombectomy

eFigure. Monthly Rates From April to December 2020 of Patients With Ischemic Stroke and Comorbid COVID-19 and Their Rates of In-Hospital Death

This supplementary material has been provided by the authors to give readers additional information about their work.

© 2021 de Havenon A et al. *JAMA Network Open*.

eTable 1. *International Classification of Diseases, Tenth Revision, Procedure Coding System* (*ICD-10-PCS*) Codes Used for Identification of Selected Study Variables

**Identifier**	**Variable**	**Code**
ICD-10-PCS	Endovascular thrombectomy for acute ischemic stroke[1]	03CG3Z7, 03CG3ZZ, 03CG4ZZ, 03CK3Z7, 03CK3ZZ, 03CL3Z7, 03CL3ZZ, 03CM3Z7, 03CM3ZZ, 03CN3Z7, 03CN3ZZ
ICD-10-PCS	Acute ischemic stroke with use of thrombolytic	3E03317

Reference for eTable 1

1. Mechanical Embolectomy for Treatment of Acute Stroke. https://www.unicare.com/dam/medpolicies/unicare/active/policies/mp_pw_a053520.html.

© 2021 de Havenon A et al. *JAMA Network Open*.

eTable 2. Monthly Counts of Patients With Ischemic Stroke and Number Treated With Intravenous Alteplase and Endovascular Thrombectomy

Date	Monthly totals of ischemic stroke discharges	Three month average of total ischemic stroke discharges	Treated with tPA	Treated with EVT
Jan-19	13961	13516	951	853
Feb-19	12547		868	752
Mar-19	14039		993	822
Apr-19	13690	13930	959	844
May-19	14565		1057	929
Jun-19	13535		939	852
Jul-19	14029	13715	1047	885
Aug-19	13963		1014	896
Sep-19	13152		972	897
Oct-19	14185	14144	1027	898
Nov-19	13803		959	950
Dec-19	14443		1061	1023
Jan-20	14739	13722	1108	985
Feb-20	13414		982	936
Mar-20	13013		977	976
Apr-20	10333	11945	736	858
May-20	12353		843	815
Jun-20	13149		866	889
Jul-20	13639	13489	939	878
Aug-20	13142		870	875
Sep-20	13682		947	952
Oct-20	13842	13546	931	882
Nov-20	12716		899	855
Dec-20	14079		924	970

Abbreviations: EVT, endovascular thrombectomy; tPA, intravenous alteplase.

© 2021 de Havenon A et al. *JAMA Network Open*.

eFigure. Monthly Rates From April to December 2020 of Patients With Ischemic Stroke and Comorbid COVID-19 and Their Rates of In-Hospital Death

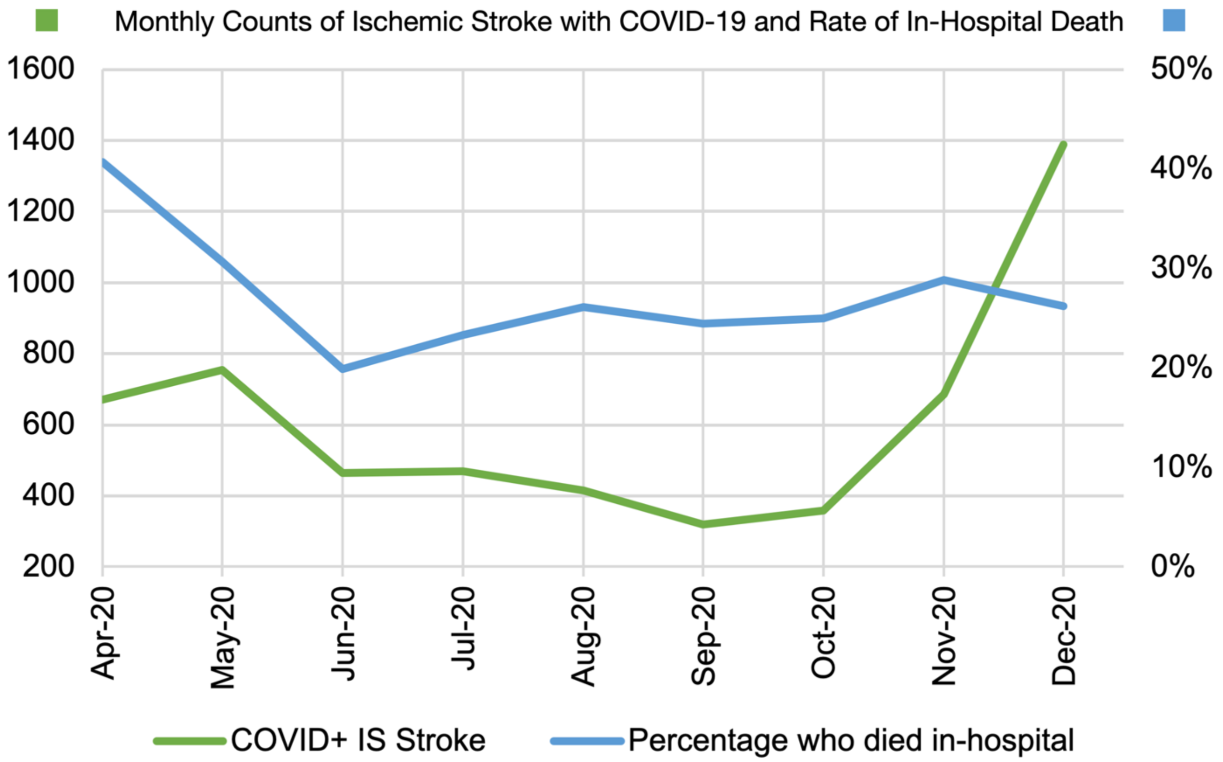

© 2021 de Havenon A et al. *JAMA Network Open*.
